# The molecular identity of the characean OH^−^ transporter: a candidate related to the SLC4 family of animal pH regulators

**DOI:** 10.1007/s00709-021-01677-3

**Published:** 2021-07-07

**Authors:** Bianca N. Quade, Mark D. Parker, Marion C. Hoepflinger, Shaunna Phipps, Mary A. Bisson, Ilse Foissner, Mary J. Beilby

**Affiliations:** 1 Department of Physiology and Biophysics, The State University of New York: The University at Buffalo, Buffalo, NY USA; 2 Department of Biosciences, University of Salzburg, Hellbrunner Str. 34, 5020 Salzburg, Austria; 3 Department of Biological Sciences and Program in Evolution, Ecology, and Behavior, The State University of New York: The University at Buffalo, Hochstetter 623, Buffalo, NY USA; 4 School of Physics, The University of NSW, Kensington, Sydney, NSW 2052 Australia

**Keywords:** *Chara*, pH banding, OH^−^ transporter, Molecular sequence, *Xenopus* oocyte system, Electrophysiology

## Abstract

Characeae are closely related to the ancient algal ancestors of all land plants. The long characean cells display a pH banding pattern to facilitate inorganic carbon import in the acid zones for photosynthetic efficiency. The excess OH^−^, generated in the cytoplasm after CO_2_ is taken into the chloroplasts, is disposed of in the alkaline band. To identify the transporter responsible, we searched the *Chara australis* transcriptome for homologues of mouse Slc4a11, which functions as OH^−^/H^+^ transporter. We found a single Slc4-like sequence CL5060.2 (named CaSLOT). When CaSLOT was expressed in *Xenopus* oocytes, an increase in membrane conductance and hyperpolarization of resting potential difference (PD) was observed with external pH increase to 9.5. These features recall the behavior of Slc4a11 in oocytes and are consistent with the action of a pH-dependent OH^−^/H^+^ conductance. The large scatter in the data might reflect intrinsic variability of CaSLOT transporter activation, inefficient expression in the oocyte due to evolutionary distance between ancient algae and frogs, or absence of putative activating factor present in *Chara* cytoplasm. CaSLOT homologues were found in chlorophyte and charophyte algae, but surprisingly not in related charophytes Zygnematophyceae or Coleochaetophyceae.

**Supplementary Information:**

The online version contains supplementary material available at 10.1007/s00709-021-01677-3.

## Introduction

**Fig. 1:**
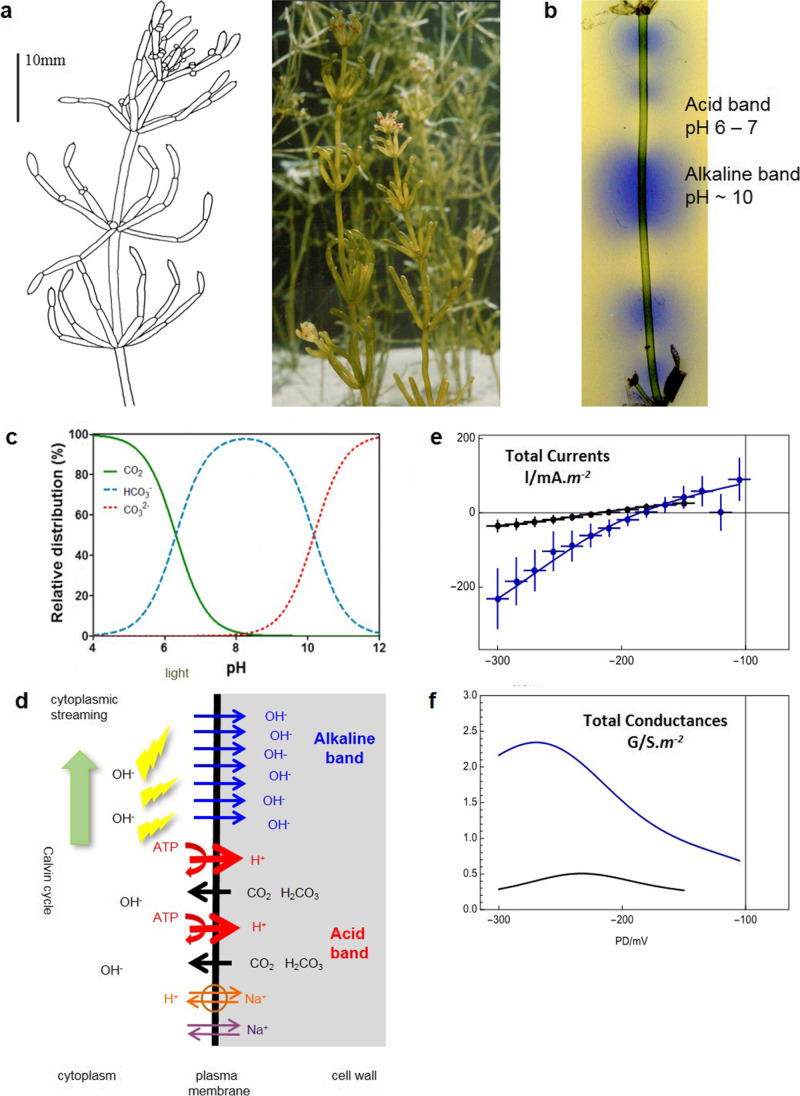
OH^−^ transporter background. **a** *Chara australis* plant (Beilby and Casanova 2014). **b** Internodal *Chara* cell in the light shows the banding pattern in pH indicator Bromothymol Blue. **c** DIC (dissolved inorganic carbon) speciation in water (Pedersen et al. 2013). **d** The transporters involved in the banding pattern and the OH^−^ transporter response in saline media (Absolonova et al. 2018). In the alkaline band: OH^−^ transporters (blue). In the acid band: H^+^ ATPase AHA (red), Na^+^/H^+^ antiporter NHX (orange), non-selective cation channel HKT (violet), which is thought to let Na^+^ into the cell in saline media (see Phipps et al. 2021, for characterization of the transporters). CO_2_ and H_2_CO_3_ (black) permeate through the lipid bilayer. For further details see text. The contrast of the **e** I/V and **f** G/V characteristics of the proton pump dominated state (black, pH 7.1, statistics: 12 I/V profiles from 5 cells) and OH^−^ transpoter dominated state (blue, pH 11.1, statistics: 12 I/V profiles from the same 5 cells). The data were fitted (lines) by Gradmann-Sanders-Slayman model for the proton pump and E-GHK model for the OH^−^ transporter (see Beilby and Bisson 2012 for model parameters)

Characeae are closely related to ancient algal ancestors of all land plants (Nishiyama 2018). Their large cells and relatively simple body plan (Fig. 1a, b) provide an excellent experimental system for research into plant physiology, electrophysiology and biochemistry on cellular and subcellular level (Hope and Walker 1975; Shimmen et al. 1994; Beilby and Casanova 2014).

To grow and to produce organic compounds, plants need to fix carbon. Many of the Characeae live in fresh water ponds and rivers with pH of 8 and above, where the dissolved inorganic carbon (DIC) takes the form of bicarbonate HCO_3_^−^ (Fig. 1c). In the light the photosynthesizing characean cells display intricate pH banding patterns (Fig. 1b). The proton ATPases (AHA) pump H^+^ out of the cell in the acid bands, generating an electrochemical H^+^ gradient and acidifying the immediate cell exterior to pH 6–7 (depending on bulk medium pH, pH_o_), converting DIC to CO_2_. This biophysical CO_2_ concentrating mechanism (CCM, Raven and Hurd 2012) facilitates inorganic carbon import in the acid zones as lipophilic CO_2_ (or H_2_CO_3_), which can move into the cytoplasm by diffusion across plasma membrane (Fig. 1d; Gutknecht et al. 1977; Walker et al. 1980). Alternatively (or in addition), HCO_3_^−^ might enter the cytoplasm by H^+^ symport or OH^−^ antiport. However, as the photosynthesis utilizes carbon in the form of CO_2_, either process generates OH^−^, increasing the cytoplasmic pH (see Fig. 11.6 of Beilby and Bisson 2012).

What happens in the alkaline band? Bisson and Walker (1980, 1981) increased the pH_o_ to levels between 9 and 12. The whole cell became an alkaline band and the electrophysiology of the dominant transporter could be investigated. The membrane potential difference (PD) followed the equilibrium PD for OH^−^/H^+^ (E_OH_ = E_H_) with rising pH, hyperpolarizing by − 59 mV/pH unit (like a pH electrode) to more than − 200 mV at pH_o_ 12. The shape of the current/voltage (I/V) characteristics acquired downward curvature (Fig. 1e), and the membrane conductance increased (Fig. 1f), in some cells up to 10 S.m^−2^. Beilby and Bisson (1992; 2012) analysed the I/V characteristics in detail, using the Goldmann-Hodgkin-Katz (GHK) equation for OH^−^ or H^+^, multiplied by the Boltzmann distribution of open probabilities, to model stronger PD-dependence than provided by GHK alone (enhanced GHK: E-GHK; see Fig. 1e, f; Amtmann and Sanders 1999; Beilby and Walker 1996; Beilby et al. 2017). The large conductance and tendency of the membrane PD toward E_OH_ suggest an OH^−^ channel. However, until the single channel properties can be characterized, we will use the term “OH^−^ transporter”.

Do the transporters mediate an efflux of OH^−^ or influx of H^+^? The substitution of H^+^ or OH^−^ into the calculation leads to the same I/V characteristics. However, as the concentration of H^+^ in the medium drops with the rising pH, the N_H_P_H_ parameter in the equation (the number of transporters multiplied by the transporter permeability) has to be increased by many orders of magnitude to account for the experimental currents and conductances. On the other hand, N_OH_P_OH_ parameter does not need adjustment to model the observed I/V characteristics throughout the pH_o_ range (Beilby and Al Khazaaly 2009; Beilby and Bisson 2012). Thus the modelling suggests an OH^−^ transporter (Fig. 1d).

How are the OH^−^ transporters activated at high pH_o_ and in the alkaline band? The cytoplasmic pH, pH_cyt_ is tightly regulated between pH_o_ 5.5 and 7.5, but at higher levels the pH_cyt_ becomes more dependent on pH_o_ (Smith 1984). When the pH_cyt_ reaches some critical point, the OH^−^ transporters activate (Bisson and Walker 1982). In the alkaline band, the increase in OH^−^ results from assimilation of CO_2_ in the chloroplasts performing photosynthesis in the light. Lucas and Shimmen (1981) and later Bulychev and Dodonova (2011) suggested that there might also be messengers, generated by the illuminated cytoplasm, that affect the cell downstream, including OH^−^ transporter activation. However, the identity of such messengers remains unknown.

The pH banding system is transiently suppressed by passage of an action potential. The conductance in alkaline zones decreases, with recovery between 15–30 min (Bulychev and Krupenina 2009, Dodonova et al. 2010, for review see Beilby and Bisson 2012). Smith and Beilby (1983) measured transient decrease of conductance after an action potential (averaged over the whole cell). In some experiments the media pH was buffered, which would inhibit the banding process. They suggested proton pump inhibition. More experiments are needed to find the mechanisms involved.

Under normal conditions, the OH^−^ transporter is an important element of the CCM function and efficient photosynthesis. However, in saline media these transporters become part of the pathology, limiting the survival of the salt sensitive *Chara australis* in salinity as low as 50–100 mM NaCl. Upon exposure to saline, the membrane PD develops typical noise (Al Khazaaly et al. 2009) and the banding pattern transforms into transient alkaline spots (Absolonova et al. 2018). After hours in the saline medium, the membrane PD depolarizes due to inhibition of the H^+^ ATPase (Shepherd et al. 2008), the lifetime of the alkaline spots increases (see Fig. 4 of Absolonova et al. 2018), the noise amplitude diminishes (see Al Khazaaly et al. 2009 for detailed noise analysis), and the upwardly concave I/V characteristics can be modelled by OH^−^ and E-GHK (for E-GHK parameters see Table 5 of Al Khazaaly and Beilby 2012). The activation of OH^−^ transporters by saline media allowed Beilby and Al Khazaaly (2009) to investigate the PD-dependence of the transporters over greater pH_o_ range (7–12). At pH_o_ 7 the OH^−^ transporters tend to open at more positive PDs. Consequently, the initial activation of small groups of OH^−^ transporters, while the membrane PD was still negative, resulted in transient activation: transient alkaline spots. As the membrane PD becomes depolarized with saline exposure, the OH^−^ transporters remain active and start to dominate the membrane conductance, moving the membrane PD towards 0 and eroding the H^+^ electrochemical gradient that drives many transporters, including the Na^+^/H^+^ antiport. The cell has reached a point of no return (Shepherd et al. 2008). However, the salt-tolerant Characeae, *Lamprothamnium*, exhibits pH banding in salinities as high as seawater (Foissner unpublished; Bisson unpublished).

What activates the OH^−^ transporters at low pH_o_ of 7? Reactive oxygen species (ROS) signalling might be involved (Eremin et al. 2013; Dodonova et al. 2010; Li et al. 2007). Pre-treatment of cells in antioxidant melatonin postponed the membrane PD noise upon the exposure to saline (Beilby et al. 2014). The possibility that the hydroxyl radical might activate the channels needs to be investigated.

Are the properties of Characeae OH^−^ transporters relevant to land plants in general? The pH banding motif can be found in aquatic angiosperms (Prins et al. 1980). Further, land plant roots band to improve molybdenum, phosphorus, and iron acquisition and reduce aluminium toxicity. OH^−^/H^+^ transporters are found near the root tip, while acid band is in the subapical zone (Raven 1991). So there is a possible role of the OH^−^ transporters in efficient function of land plants and in salt sensitivity/tolerance.

Al Khazaaly and Beilby (2012) were inspired by experiments on the voltage-gated H^+^ channel Hv1 and applied 1 mM Zn^2+^ — a powerful blocker of Hv1 (DeCoursey and Hosler 2014). The pH banding, the membrane PD noise, and the typical OH^−^ I/V characteristics at the time of saline stress were all abolished. However, as the *Chara* genome became available (Nishiyama et al. 2018), no homology to animal proton channels was reported (Hoepflinger, unpublished). We communicated with animal proton channel expert Thomas DeCoursey, who suggested that a member of the HCO_3_^−^ transporter family, Slc4a11, which was shown to transport H^+^ or OH^−^ in animal systems (Myers et al. 2016; Kao et al. 2015), may be responsible. This paper describes the experiments to clone an Slc4a11-like gene from *Chara* and to examine its OH^−^ transporting function when expressed in *Xenopus* oocytes.

## Material and methods

### Algal material and culture conditions

*Chara australis* thalli were grown in 10–50 L aquaria in a substrate of soil, peat, and sand filled with distilled water. Fluorescent lamps provided a 14/10 h light/dark cycle at a temperature of about 20 °C. To prevent calcification as well as growth of epiphytes, light intensity was low (about 5 μM.m^−2^.s^−1^).

### Cloning and sequence analyses

The transcriptomic database of *Chara australis* described in Pertl-Obermeyer et al., 2018 (EBI-ENA data base accession no. ERP023711) was screened for homologous proteins of mouse Slc4a11 (Myers et al. 2016). In order to verify the obtained sequence, fresh total RNA was extracted from *C. australis* thalli using TRI-Reagent according to manufacturer’s instructions (Sigma-Aldrich). Residual genomic DNA was digested by RNase-free DNase (EN0521, Thermo Fisher Scientific, Waltham, MA, USA). First-strand cDNA was synthesized from 1 μg total RNA by M-MuLV Reverse Transcriptase (RevertAid; EP0441, Thermo Fisher Scientific) and an anchored oligo(d)T primer-mix according to the supplier’s protocol. The obtained cDNA was used as template for PCR amplification with Phusion High-Fidelity DNA polymerase (F530S, Thermo Fisher Scientific) also according to manufacturer’s instructions and the following primers: 5’-GCCCGGGATGCGTACGCATGCAATGAG-3’ (including XmaI restriction enzyme site), 5’-GTCTAGATCAATGCGCATCAAGGACTCTCAG-3’ (including XbaI restriction enzyme site). The obtained amplicon was sub-cloned into pJet1.2 cloning vector (Thermo Fisher Scientific, #K1231), digested with XmaI and XbaI, ligated into pGH19 expression vector which includes sequence that encodes a poly(A) tail (Musa-Aziz et al. 2010) and verified by sequencing (Eurofins Genomics LLC, Louisville, KY)*.* CL5060.2 we called CaSLOT (*Chara* *australis* SLC4-like *O*H(H) transporter protein; accession number: MN103545).

Annotated plant and animal sequences were used for BLAST analyses (Altschul et al. 1990) in order to reveal sequences that are homologous to our *C. australis* SLOT protein. These sequences were aligned using ClustalW or Clustal Omega (for two or more sequences, respectively; EMBL-EBI). Conserved domains were detected by InterPro (Mitchell 2015) as well as the conserved domain search tool of NCBI (Marchler-Bauer et al. 2015).

### Oocyte preparation

*Xenopus laevis* (Xenopus Express Inc., Brooksville, FL) frogs were handled in compliance with the protocols approved by the University at Buffalo Institutional Animal Care and Use Committee. Ovaries were harvested from frogs anesthetized with 0.2% tricaine. Ovary fragments were washed with sterile calcium-free Ringer solution (Ca-free NRS: 82 mM NaCl, 20 mM MgCl_2_, 5 mM HEPES, and 2 mM KCl, pH 7.50) for 5 min, 3 times. The fragments were treated with Ca-free NRS solution with 2 mg/ml type-1A collagenase (Sigma-Aldrich, St. Louis, MO) and allowed to mix until the follicular layer of the oocytes began to dissociate. Oocytes were rinsed in Ca-free NRS for 10 min, 3 times; ND96 (96 mM NaCl, 2 mM KCl, 1.8 mM CaCl_2_, 1 mM MgCl_2_, 5 mM HEPES, pH 7.50) for 10 min; and OR3 for 10 min (OR3: 14 g Leibovitz’s L-15 medium powder (Thermo Fisher Scientific), 5 mM HEPES, 20 mL 100X penicillin–streptomycin (Corning Inc., Corning, NY), pH 7.5, 200 mosmol/kgH_2_O). Cells were sorted and stored at 18 °C in fresh OR3.

### cRNA preparation and injection

CaSLOT.pGH19 cDNA was digested with NotI and purified with the MinElute PCR Purification kit (Qiagen). Linearized DNA was used as a template to produce capped cRNA with the T7 mMessage mMachine Transcription kit (Invitrogen, Carlsbad, CA). cRNA was purified with the RNeasy MinElute Cleanup kit (Qiagen), and quantified using a NanoDrop 2000 (Thermo Fisher Scientific) before injection into oocytes (15 ng/oocyte) with a Nanoject III programmable injector (Drummond Scientific Co., Broomhall, PA).

### Electrophysiology

Oocytes were superfused in a small chamber (no.RC-3Z, Warner Instruments, Hamden, CT) on an antivibration worktable (Vision IsoStation, Newport Corp., Irvine, CA). Solutions were fed at 2 mL/min from syringe pumps (Harvard Apparatus, Holliston, MA). Bath solutions were based around ND96 (93.5 mM NaCl, 2 mM KCl, 1.8 mM CaCl_2_, 1 mM MgCl_2_, and 5 mM of buffer). The pH 7.5 solution was buffered with HEPES, while our pH 9.5 solution was buffered with CHES. Microelectrodes were pulled using a P-1000 micropipette puller (Sutter Instrument, Novato, CA) from borosilicate glass (no. BF200-156–10, Sutter Instrument). The electrodes were filled with saturated KCl solution and had a tip resistance of 0.1–2 MΩ. Voltage and current electrodes connected to an OC275 oocyte clamp (Warner Instruments, Hamden, CT) were used to impale oocytes. A bath electrode (no. 7251, Warner Instruments) was used to clamp the bath potential to 0 mV. Oocyte membrane current–voltage (I–V) relationships were gathered by clamping the membrane potential (*V*_m_) to its resting potential and from – 160 mV to 0 mV in 20 mV steps for 100 ms, retuning to resting potential for 100 ms in between each step. I–V data were acquired using a Digidata 1550 with Clampex 10.4 software (Molecular Devices LLC, San Jose, CA). I–V plots were graphed in Microsoft Excel and membrane conductance (*G*_m_) was calculated as the slope of the trace.

## Results

### Identification and protein sequence analyses of CaSLOT

In order to detect proteins comparable to voltage-gated H^+^ channels in the green alga *Chara australis*, we aligned different plant sequences that had been described to exhibit this transport function. As we were not able to find any homologous proteins in our *Chara australis* dataset, we tested sequences from the animal kingdom: mouse Slc4a11 transporter was described as a pH-sensitive OH^−^/H^+^ transporter in 2016 by Myers et al. (2016). When compared to our *C. australis* transcriptomic dataset, a single Slc4-like sequence was found: CL5060.Contig2 (or CL5060.2). Following sequence as well as functional analyses we named this sequence CaSLOT (SLC4-like OH(H) transporter protein; accession number: MN103545).

**Fig. 2:**
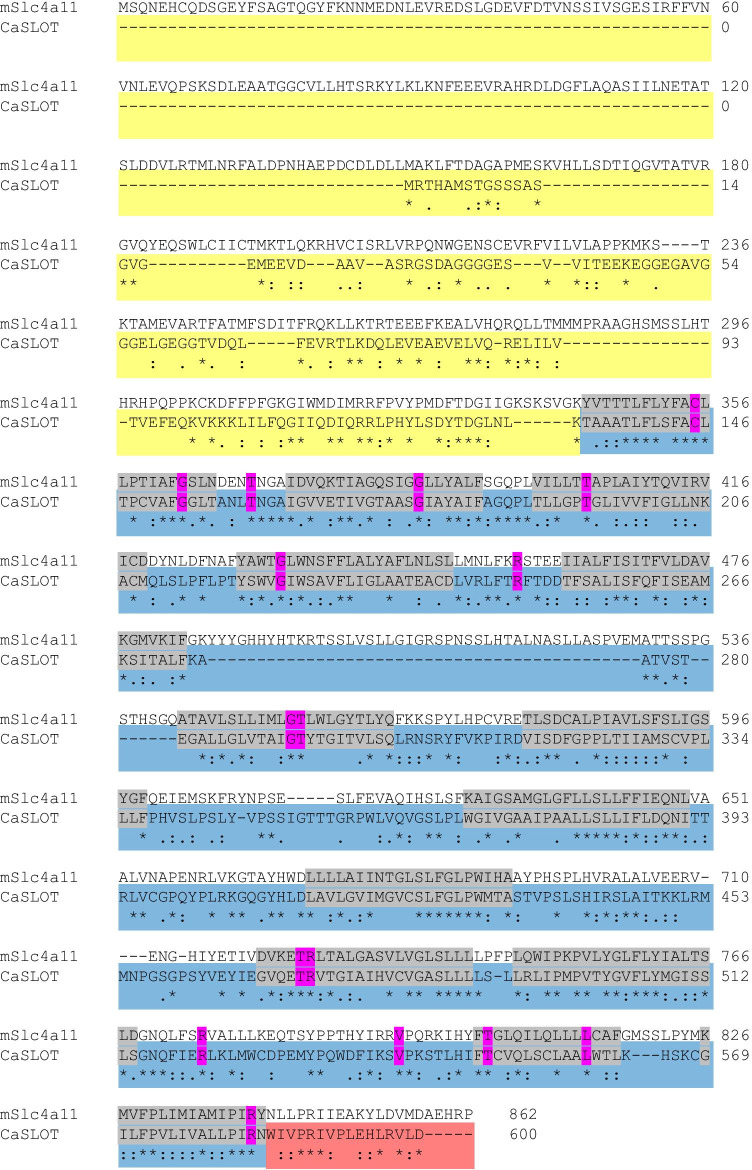
Protein sequence alignment of mouse Slc4a11 and *Chara australis* CaSLOT. Alignment performed by Clustal Omega (https://www.ebi.ac.uk/Tools/msa/clustalo/) and manually refined. Colours represent the three major structural domains: cytosolic amino-terminal domain (yellow), membrane-spanning domain (blue, with individual membrane spans in grey), and cytosolic carboxy-terminal domain (red). Conserved residues mutated in disease in human SLC4A11 (Alka and Casey 2018) are shown in pink

CaSLOT encodes a protein consisting of 602 amino acids and a calculated molecular weight of 64.75 kDa. We had a closer look at the similarities and differences of the protein sequences of CaSLOT and mouse Slc4a11. As seen in Fig. 2, CaSLOT lacks the large structured cytosolic Nt domain of Slc4a11 and also lacks the extended extracellular loop between the 5th and 6th membrane spanning helices. Between their membrane domains, CaSLOT and mouse Slc4a11 share 34% identity at the amino acid level. Moreover, 16 of the 30 conserved residues within the membrane domain of SLC4A11 that are mutated in human disease (Alka and Casey 2018) are preserved in CaSLOT (mutation spots are marked in pink in Fig. 2). However, Slc4a11 is a vertebrate gene designation, and sequence alignments of CaSLOT with other SLC4 and Slc4-like (i.e., non-vertebrate) proteins suggest that CaSLOT is generally Slc4-like rather than being specifically Slc4a11-like. Using the criteria defined by Parker and Boron (see Fig. 4 in Parker and Boron 2013), CaSLOT would be defined as “Primitive” bearing more identity (48%) to the functionally uncharacterized Slc4-like protein from the nitrifying bacterium *Nitrococcus mobilis* than the archetypal Slc4-like boron transport protein (AtBOR1, 39%) from land plants or to any Slc4 or Slc4-like proteins from the fungal or animal kingdoms.

### CaSLOT compared to other algae

**Fig. 3:**
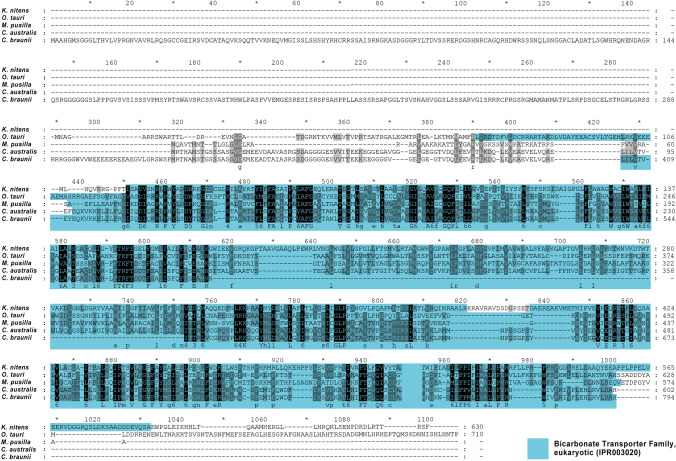
Protein sequence alignment of homologous forms of CaSLOT proteins of various green algae. Bicarbonate transporter family domains (IPR003020) are marked according to InterPro analyses (https://www.ebi.ac.uk/) and denote sequence homology to bicarbonate transporters of the SLC4 protein family rather than demonstrated functionality. Sequences used in the alignment: *Chara australis* SLOT (CaSLOT, GenBank: MN103545); *Chara braunii*, hypothetical protein CBR_g23541 (GenBank: GBG77214.1); *Klebsormidium nitens* (GenBank: GAQ87198.1); *Ostreococcus* tauri, HCO_3_^−^ transporter family-domain-containing protein (GenBank: OUS47809.1); *Micromonas pusilla* (XP_003055831.1)

We compared CaSLOT via  BLAST analyses (Altschul et al. 1990) to other green algae and detected several homologous proteins in Klebsormidiophyta (*Klebsormidium nitens*), Charophyta (*Chara australis* and *Chara braunii*), and Chlorophyta (*Ostreococcus tauri, Micromonas pusilla*). Surprisingly, there were no homologous proteins of CaSLOT detectable in Coleochaetophyceae and Zygnematophyceae, both classes of the charophyte algae as well. According to Interpro, all homologous proteins contain one or more bicarbonate transporter family domains in various lengths throughout the sequences that showed nice homologies (see Fig. 3): CaSLOT showed 22% identical amino acids when compared to *K. nitens*; 27% to *O. tauri* and 33% to *M. pusilla.*

When compared to the sequenced genome of *Chara braunii,* CaSLOT showed homologies to two *C. braunii* proteins: CBR_g23541 (hypothetical protein, GenBank: GBG77214.1) and CBR_g66734 (hypothetical protein, GenBank: GBG59928.1). Direct comparison revealed 50% identical amino acids between CaSLOT and *C. braunii* CBR_g23541. CaSLOT and CBR_g66734 showed only 11% identical amino acids. Both *C. braunii* sequences compared to each other showed only 15% identical amino acids. Compared to CaSLOT the *C. braunii* sequence has an N-terminal elongation of 317 amino acids and lacks 121 amino acids in the middle of the bicarbonate transport domain part (see Fig. 3) a region that encompasses transmembrane spans 6–8. A protein sequence alignment of CaSLOT, CBR_g23541, and mouse Slc4a11 revealed that CaSLOT shows a slightly higher homology to Slc4a11 (25% identical amino acids) than the *C. braunii* sequence (22% identical amino acids). According to our analyses this N-terminal stretch of CBR_g23541 is only detectable in *C. braunii*. When aligned to all available databases of green plants (blast analyses), the 317 amino acids of the N-terminus revealed no homologous sequences. As the *C. braunii* sequence is not cloned and verified by sequencing at present, this stretch and the missing part in the bicarbonate transporter domain are possibly artefacts of sequence assembly during genome sequencing. The shorter N-termini of all other compared algal sequences showed no defined domains but are either predicted to be cytoplasmic (*C. australis* and *braunii*, *M. pusilla*) or non-cytoplasmic regions (*K. nitens, O. tauri*).

The functions of these Slc4-like proteins are unknown, although it is possible that some may share the boron transport function of Slc4-like proteins (BORs) from land plants (Parker and Boron 2013). *Chara* also exhibits boron transport (Stangoulis et al. 2001), and it will be interesting if the Slc4-like transporter is involved.

### CaSLOT compared to land plants

**Fig. 4:**
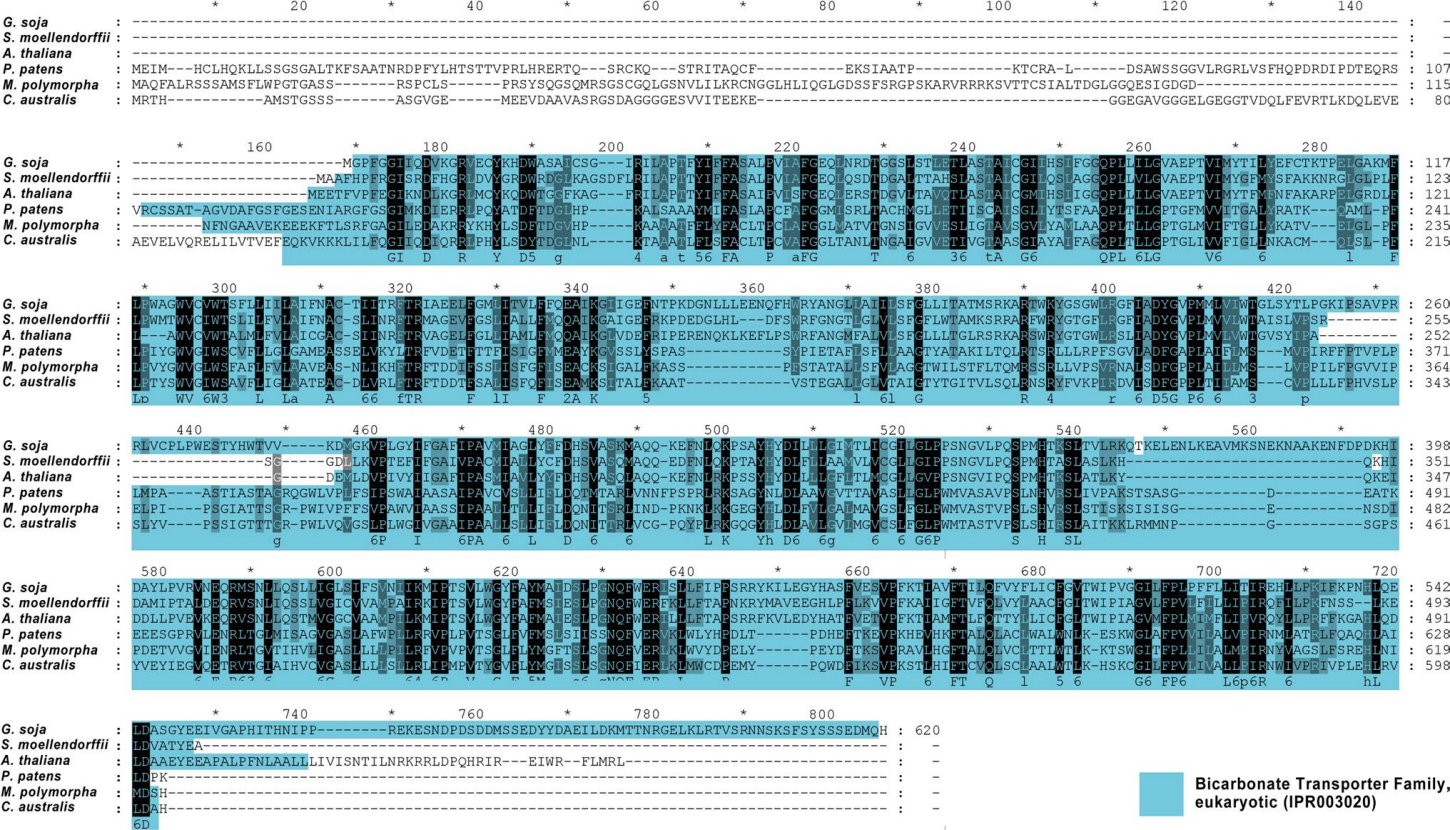
Protein sequence alignment of homologous forms of CaSLOT proteins of various land plants. Bicarbonate transporter family domains (IPR003020) are marked according to InterPro analyses (https://www.ebi.ac.uk/) and denote sequence homology to bicarbonate transporters of the SLC4 family rather than functionality. Sequences used in the alignment: *Arabidopsis thaliana* (GenBank: AAD26598.1); *Glycine soja* (GenBank: KHN06659.1); *Selaginella moellendorffii*, hypothetical protein (GenBank: EFJ22813.1); *Physcomitrella patens*, anion exchange protein 4-like isoform X1 (XP_024395568.1); *Marchantia polymorpha*, hypothetical protein (GenBank: PTQ38262.1); and *Chara australis* SLOT (CaSLOT, GenBank: MN103545)

An alignment of CaSLOT against various homologous sequences from land plants can be seen in the protein alignment of Fig. 4. At present, with the exception of the *A. thaliana* protein, which is a boron transporter, the functions of these proteins have not been analysed. As seen in Fig. 4, the bicarbonate transporter domains reveal good homologies. *P. patens* and *M. polymorpha* show elongated N-terminal stretches, predicted to be cytoplasmic. The same is true for CaSLOT. However, *S. moellendorffii*, *G. soja*, and *A. thaliana* do not show this elongated N-terminus. Instead, the region of the bicarbonate transporter family domain starts already at amino acid 2–3. (Overall, the calculated homologies — identical amino acids to CaSLOT: vs *M. polymorpha*: 51%; vs *P. patens*: 42%; vs *S. moellendorfii*: 25%; vs *G. soja*: 22%; vs *A. thaliana*: 23%).

### Electrophysiological study of CaSLOT expressed in Xenopus oocytes

**Fig. 5:**
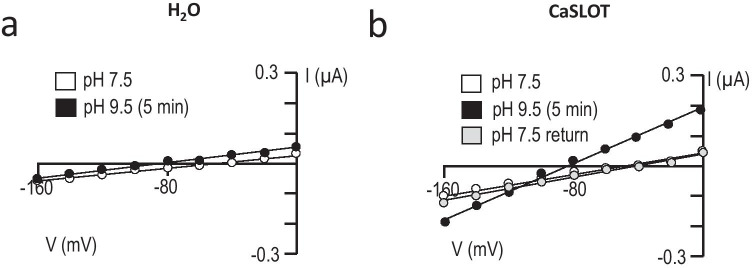
Representative I-V plots gathered from H_2_O-injected and CaSLOT-expressing oocytes. **a** H_2_O-injected cell and **b** CaSLOT-cRNA-injected cell. The white data points show the initial relationship between current and voltage with the cells in pH 7.5 bath solution. The black data points show the relationship between current and voltage of the cell membrane after 5 min of exposure to pH 9.5 bath solution. The grey data points in panel **b** show that the change in the relationship between current and voltage in CaSLOT-expressing cells in response to increased extracellular pH is reversible upon return to pH 7.5 bath solution

**Fig. 6:**
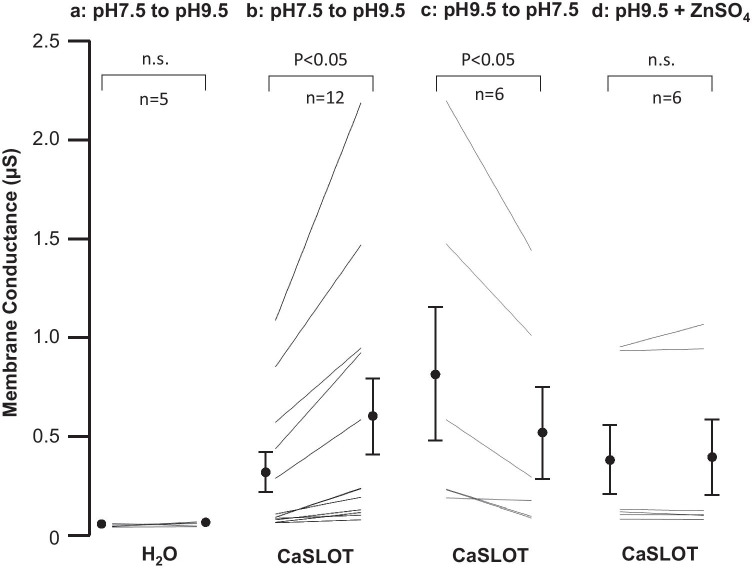
Comparison of conductance values gathered from water-injected controls and CaSLOT-expressing oocytes. The first column (**a**) shows the response of H_2_O-injected cells as the bath solution switches from pH 7.5 to pH 9.5. The second column (**b**) shows changes in conductance of CaSLOT-expressing cells under the same conditions. The third column (**c**) shows the effect on a subset of the cells from column (**b**), of restoring bath pH to 7.5. The fourth column (**d**) shows how the conductance of a subset of the CaSLOT-expressing cells from column (**b**) responds to a 5 min exposure to 1 mM ZnSO_4_. Statistics are the result of paired t-tests

Figure 5a shows a pair of representative current–voltage relationships (I–V plots) obtained from a H_2_O-injected oocyte as it was superfused either with a pH 7.5 solution (white circles) or a pH 9.5 solution (back circles) for 5 min. In five such replicates, the switch to the more alkaline solution caused a significant hyperpolarizing shift in membrane potential from – 45 ± 1 mV to – 73 ± 4 mV (P < 0.05, one-tailed, paired t-test: an average delta *V*_m_ of – 28 ± 3 mV), but no significant alteration in membrane conductance (*G*_m_, see Fig. 6a).

Figure 5b shows a series of representative I–V plots obtained from an oocyte that had been injected with CaSLOT cRNA 3 days prior, as the cell was sequentially superfused with a pH 7.5 solution (white circles), a pH 9.5 solution (black circles), and finally returned to the original pH 7.5 solution (grey circles). Considering a larger number of cells, at the start of the experiment in the pH 7.5 solution, the *V*_m_ of CaSLOT-cRNA-injected cells (– 35 ± 4 mV, n = 12) was not significantly different from the *V*_m_ of H_2_O-injected cells (P > 0.05, two-tailed, unpaired t-test). The *G*_m_ of CaSLOT-cRNA-injected cells at pH 7.5 tended to be greater on average than that of H_2_O-injected cells (see Fig. 6a versus Fig. 6b, shown on expanded axes in Supplemental Fig. 1), but the difference was not significant (P > 0.05, two-tailed, unpaired t-test). Upon exposure to pH 9.5 solution, CaSLOT-cRNA-injected oocytes exhibited a significant hyperpolarization to – 78 ± 4 mV (P < 0.05, one-tailed, paired t-test: an average delta *V*_m_ of – 43 ± 4 mV). This represents a significantly greater hyperpolarization than that exhibited by H_2_O-injected cells in response to pH 9.5 solution (P < 0.05, one-tailed, unpaired t-test). In CaSLOT-cRNA-injected cells, this hyperpolarization was accompanied by a significant increase in *G*_m_ (Fig. 6b). Despite a wide variation in the magnitude of increase, the fractional increase in *G*_m_ was more consistent (1.8 ± 0.1 fold-change).

Six of the 12 CaSLOT-cRNA-injected cells that had been exposed to pH 9.5 solution were subsequently returned to pH 7.5 solution, as shown in the representative example in Fig. 5b. In these six cells, *V*_m_ (not shown but exemplified in Fig. 5b) and *G*_m_ (Fig. 6c) returned to their pre-pH-9.5-solution-exposure values (P > 0.05, two-tailed, paired t-test comparing these parameters during the original and “post-pH-9.5” pH-7.5 solution exposure).

The remaining six of the 12 CaSLOT-cRNA-injected cells that had been exposed to pH 9.5 solution were exposed to a pH 9.5 solution containing 1 mM ZnSO_4_. Although this manoeuvre did not significantly alter *G*_m_ (Fig. 6d), it did produce a significant depolarization of *V*_m_ (from – 87 ± 4 mV to – 66 ± 3 mV, P < 0.05, one-tailed, paired t-test) towards its value in pH 7.5 solution.

## Discussion

Our electrophysiological data reveal that injection of CaSLOT cRNA into *Xenopus* oocytes causes the expression of a membrane conductance that is activated by alkaline pH and which hyperpolarizes *V*_m_ (i.e., towards the predicted equilibrium potential for OH^–^). These are features shared by Slc4a11-expressing oocytes and are consistent with the expression of a pH-sensitive OH^–^/H^+^ conductance (Myers et al 2016; Quade et al. 2020). Let us first consider the pH-dependent hyperpolarization. Considering the electrochemical gradients across the oocyte membrane, such a phenomenon could either reflect the opening of a pH-sensitive K^+^ channel (*E*_K_, the equilibrium potential for K^+^ is ~ – 100 mV) or an OH^–^/H^+^ channel (*E*_H/OH_ the equilibrium potential for OH^–^/H^+^ is ~ – 120 mV) causing the oocyte *V*_m_ to shift towards the equilibrium potential for that ion. Although hyperpolarization is also an endogenous feature of H_2_O-injected oocytes exposed to pH 9.5 solution, in CaSLOT-expressing oocytes, it is exaggerated, suggesting ion permeability that is accompanied by an increase in G_m_. Considering the homology between CaSLOT and Slc4a11, we consider pH-sensitive OH^–^/H^+^ conductance to be the most likely explanation for these features of CaSLOT-expressing cells.

We note two differences between the features of CaSLOT-expressing and Slc4a11-expressing oocytes. Firstly, in CaSLOT cells, *V*_m_ does not reach *E*_H/OH_ which likely reflects a less robust expression and an inability of the heterologously expressed activity to dominate *V*_m_. Secondly, according with the previous observation, the alkalinity-induced *G*_m_ is smaller in CaSLOT cells than in Slc4a11-expressing cells by an order of magnitude. We are unable to determine how robustly CaSLOT protein expresses to the plasma membrane of oocytes in relation to Slc4a11, but being an algal protein, rather than a vertebrate protein, may be a disadvantage in terms of heterologous overexpression. We note that the codon usage of CaSLOT does not accord well with the *Xenopus* codon usage table, although injection of cRNA for a *Xenopus* codon-optimized version of CaSLOT did not result in an appreciable enhancement of measurable activity (data not shown). Furthermore CaSLOT, unlike Slc4a11, could not be coaxed to mediate greater conductance by raising intracellular pH (pH_i_, data not shown) suggesting either that the conductance mediated by CaSLOT is not as exquisitely pH_i_ sensitive as Slc4a11, that a cytoplasmic factor unique to *Chara* cytoplasm is required for the response to pH_i_, or that the CaSLOT conductance was already maximized in our expression system.

Another curious observation that perhaps also speaks to heterologous expression issues is that the *G*_m_ of CaSLOT-expressing cells was very variable between cells (see Fig. 6b). This impacted our data analysis as the large standard deviation is probably responsible for the lack of statistical significance when comparing *G*_m_ of H_2_O-injected and CaSLOT-expressing cells in pH 7.5 solution, despite *G*_m_ being five times greater in CaSLOT cells on average.

However, when investigated in the native system of the Characeae, the high pH conductance, presumably mediated by the CaSLOT transporter, also exhibits fluctuations in magnitude (see Fig. 4 of Beilby and Bisson 1992). The conductance increase activates at pH_o_ 9.5, with maxima of 4–6 S.m^−2^ in pH_o_ range 10–11.5 and a tendency to decline at pH_o_ 12 (Beilby and Bisson 1992; 2012).

With the medium delivered efficiently by fast flow, the conductance rises within seconds in response to high pH_o_ (Beilby and Bisson 1992). Intact *Chara* cells and cytoplasm-enriched fragments (Beilby and Shepherd 1989) exhibited similar high conductance activation and subsequent occasional conductance changes. To make the cytoplasm-enriched fragments, long internodal cells are centrifuged to move the flowing cytoplasm to one end and tying this end off: there is no vacuole and the fragment has a greater volume of cytoplasm than an intact cell of the same surface area (Hirono and Mitsui 1981). The fast response of high pH conductance in both experimental systems suggests that it is the pH_o_ increase that activates the transporter. However, intact cells pre-treated in low pH_o_ to lower their cytoplasmic pH needed greater pH_o_ increase to activate the high conductance (Bisson and Walker 1981). As mentioned in the “Introduction”, the alkaline band is initiated by an increase in OH^−^ concentration in the cytoplasm and/or by another cytoplasmic messenger (Bulychev and Dodonova 2011). The existence of an unknown cytoplasmic messenger is becoming more likely, as the increase of the internal oocyte pH failed to produce hyperpolarization of the membrane PD and higher conductance.

The transient changes in high pH conductance and the shape of the I/V profile (Beilby and Bisson 1992) suggest fluctuations in numbers of activated/opened CaSLOT transporters. The cause of this response needs to be investigated in future experiments.

Although Slc4a11 is not known to be Zn^2+^ sensitive, Al Khazaaly and Beilby (2012) found that 1 mM Zn^2+^ totally inhibits the *Chara* OH^–^ conductance within ~ 30 min. This effect is entirely reversible by applying 0.5 mM 2-mercaptoethanol, which suggests that Zn^2+^ is acting at a sulfhydryl group, a known activity of heavy metals including Zn^2+^ (Valko et al. 2015). There is a cysteine (C386 in human SLC4A11; Alka and Casey 2018), known to be required for activity in Slc4a11 and conserved in CaSLOT (C335C355 in mouse Slc4a11, C145, respectively; Fig. 2), but we found no evidence for significant inhibition by a 5 min exposure to Zn^2+^ in the oocyte experiments (Fig. 6 and Supplemental Fig. 1). This could be due to poor expression of CaSLOT in these particular experiments, resulting in low activity. Further experiments to try to measure Zn sensitivity in a system with improved expression could be useful, but as Zn inhibits many types of transporters, this would not uniquely characterize this transporter.

## Conclusions

We present evidence that ancestral giant-celled Characeae express a Slc4-like gene CaSLOT, which shows functional homology to the Slc4a11 OH^–^/H^+^ transporter of the vertebrate cornea (Myers et al. 2016) and is therefore a candidate for the remarkable OH^−^ transporter of Characeae. There are homologues to CaSLOT in chlorophytes and charophytes (but surprisingly not Zygnematophyceae or Coleochaetophyceae). The detailed process of the activation of the characean OH^−^ channel and of CaSLOT (by increasing the cytoplasmic pH, pH_o_ above 9, formation of hydroxyl radicals at the time of saline stress, or by unknown cytoplasmic factors) needs further investigation. Future studies should also address the generation and phenotyping of CaSLOT-deletion strains of *Chara,* as well as the location of CaSLOT protein compared to the site of alkaline banding in *Chara* cells.

## Supplementary Information

Below is the link to the electronic supplementary material.

Supplementary file1 (PDF 26.6 KB) **Fig. S1 **Magnified low conductance data (the bottom row) from Fig. 6, as H_2_O and CaSLOT injected oocytes were challenged with changes of external pH.
