# Supplementary figures and images for: The molecular identity of the characean OH− transporter: a candidate related to the SLC4 family of animal pH regulators
Source: Protoplasma. 2021 Jul 7;259(3):615–26. doi: 10.1007/s00709-021-01677-3 (PMC8738779; doi:10.1007/s00709-021-01677-3)

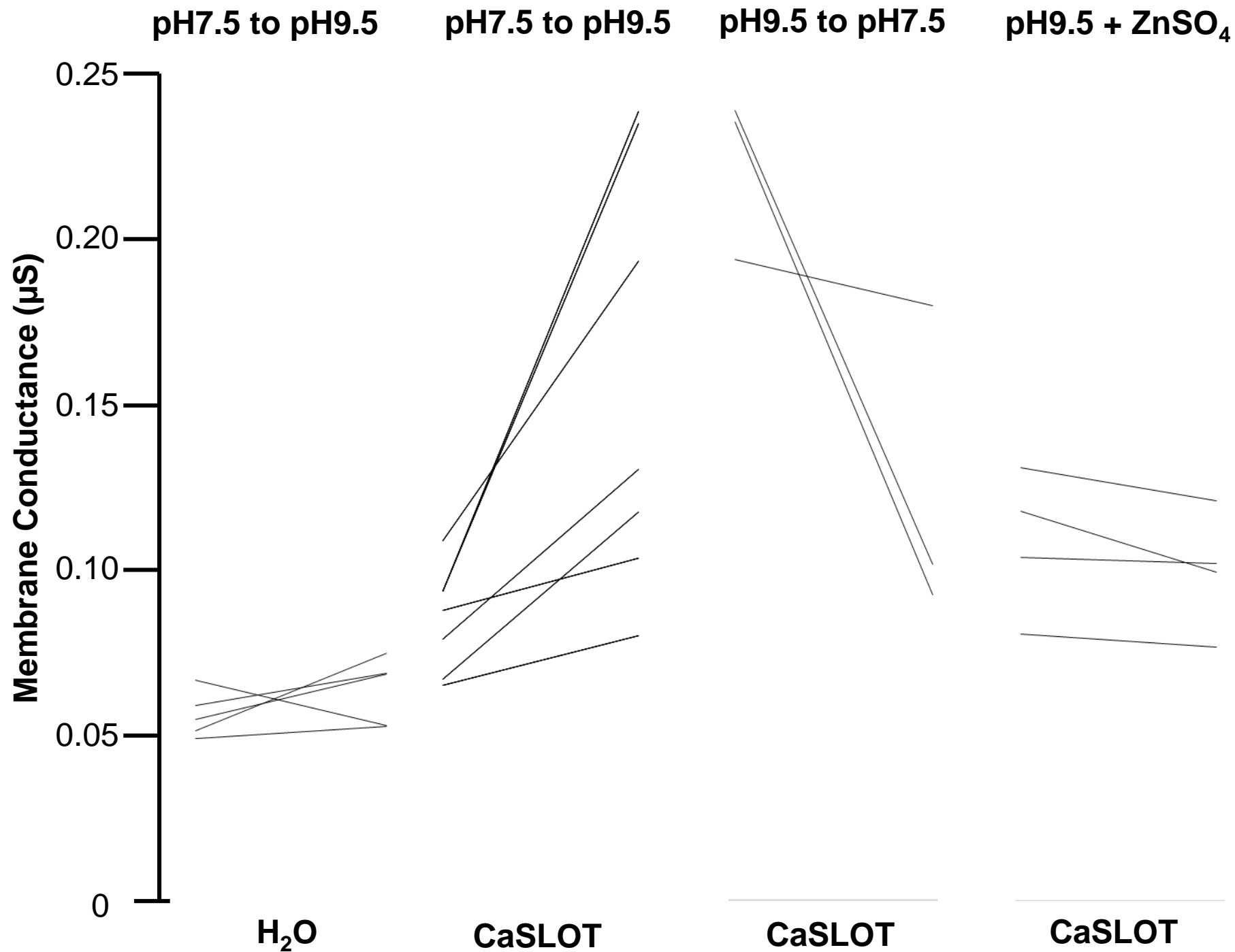

Supplement: Supplementary file 1 — Supplementary file1 (PDF 26.6 KB) Fig. S1 Magnified low conductance data (the bottom row) from Fig. 6, as H2O and CaSLOT injected oocytes were challenged with changes of external pH. [file 709_2021_1677_MOESM1_ESM.pdf]
