# Supplementary material for: The molecular identity of the characean OH− transporter: a candidate related to the SLC4 family of animal pH regulators
Source: Protoplasma. Author manuscript; Available in PMC 2022 May 1. (PMC8738779; doi:10.1007/s00709-021-01677-3)
Supplement: Supplementary file 1 [file NIHMS1761661-supplement-Supplementary_Figure_1.docx]

**Supplementary Figures**

**Supplementary Fig. 1** Magnified low conductance data (the bottom row) from Fig. 6, as H_2_O and CaSLOT injected oocytes were challenged with changes of external pH. See Fig. 6 caption for details.
